# Fatty acid comparison of four sympatric loliginid squids in the northern South China Sea: Indication for their similar feeding strategy

**DOI:** 10.1371/journal.pone.0234250

**Published:** 2020-06-11

**Authors:** Dongming Lin, Kai Zhu, Weiguo Qian, André E. Punt, Xinjun Chen

**Affiliations:** 1 College of Marine Sciences, Shanghai Ocean University, Shanghai, China; 2 The Key Laboratory of Sustainable Exploitation of Oceanic Fisheries Resources, Ministry of Education, Shanghai Ocean University, Shanghai, China; 3 National Engineering Research Center for Oceanic Fisheries, Shanghai Ocean University, Shanghai, China; 4 Key Laboratory of Oceanic Fisheries Exploration, Ministry of Agriculture and Rural Affairs, Shanghai, China; 5 Scientific Observing and Experimental Station of Oceanic Fishery Resources, Ministry of Agriculture and Rural Affairs, Shanghai, China; 6 School of Fisheries, Zhejiang Ocean University, Zhejiang, China; 7 School of Aquatic and Fishery Sciences, University of Washington, Seattle, WA, United States of America; 8 Laboratory for Marine Fisheries Science and Food Production Processes, Qingdao National Laboratory for Marine Science and Technology, Qingdao, China; Tanzania Fisheries Research Institute, UNITED REPUBLIC OF TANZANIA

## Abstract

Feeding strategies of sympatric squid species help to understand their role in marine ecosystems. Four loliginid squids, *Uroteuthis duvaucelii*, *Uroteuthis edulis*, *Uroteuthis chinensis*, and *Loliolus uyii* are the major cephalopod species in the coastal waters of the northern South China Sea, where they occur together. We investigated their feeding strategies in terms of foraging behavior and habitat use by comparing fatty acid profiles and spatial distributions. There were no significant differences in the proportions of saturated or polyunsaturated fatty acids among species. Similar findings were obtained for most individual fatty acids that made up of an average of more than 84% of total fatty acid content for each species. Substantial overlap and high similarity in the fatty acid composition were observed. However, there were no significant effects of individual size or sampling station on the fatty acid compositions. The spatial overlap analysis demonstrated that there was clear spatial segregation and habitat use among the species. Cumulatively, our results suggest that the four squids are opportunistic carnivores, unselectively foraging on similar prey items, while spatial segregation is likely a major mechanism leading to their coexistence in the northern South China Sea.

## Introduction

Species coexistence depends partly on how organisms utilize their resources and environment [1, 2]. The ability of sympatric species to exploit different parts of a niche space is essential for their coexistence if they have similar ecological requirements [3–5]. Consequently, species may adopt different strategies in terms of resource use, for example, through differences in activity patterns (e.g., temporal segregation) and habitat use [3, 6, 7], resource-abundance-mediated foraging behavior such as trophic niche contraction [4, 8, 9], or body size-related resource partitioning [10, 11].

Squids occur in almost all the world’s marine environments [12]; and most likely occupy a similar habitat throughout their lives [13, 14] or coexist seasonally [15]. Squids have been identified as among the most important organisms in marine ecosystems, not only because they act as major nutrient vectors but also because they play a key role as “bio-indicators” of environmental conditions [16–19]. Squids are important prey resources for high trophic level predators, such as large predatory fishes, seabirds and marine mammals [19–21]. On the other hand, they feed intensively on a wide spectrum of prey items including crustaceans, microneckton and fishes [19–22], and are believed to impose top-down control on low- to mid-trophic level species [19, 23, 24]. They are also cannibalistic, frequently preying on conspecifics and other squid species [15, 25, 26]. These foraging characteristics lead to complex trophic interactions [19, 27].

Investigating the feeding strategy and consequences of resource partitioning of squids is needed for a broader understanding of the dynamics of marine ecosystems. Four sympatric loliginid squids, *Uroteuthis duvaucelii*, *Uroteuthis edulis*, *Uroteuthis chinensis*, and *Loliolus uyii*, coexist in shelf waters from the western Pacific to the Indian Ocean, competing for available resources [12, 26]. Similar to other squids, these species are characterized by high growth rates and short lifespan (usually 1 year) [28–30]. The four squids play an important role in community structure and population dynamics within the shelf ecosystem where they are found [31, 32]. For example, these squids are the major cephalopod species, and seasonally dominate the regional biomass, in the coastal waters of the northern South China Sea, i.e., *U*. *duvaucelii* during autumn, and *U*. *chinensis* in summer [33]. They are also very important for the coastal fisheries on the continental shelf off Thailand, China, and Japan [26, 31, 34, 35]. However, little is known about their feeding strategies, with the exception of *U*. *chinensis* and *U*. *duvaucelii* being reported as feeding on crustaceans, fish and cephalopods in southwestern Gulf of Thailand by Islam et al. [26].

Fatty acids are vital for organelle and physiological functions [36, 37]. In cephalopods, fatty acids are essential dietary components, not only playing a critical role in energy sourcing during starvation [38, 39], but more importantly assisting the early stages of development and growth, mostly through maternal allocation to gametes during sexual maturation [40–43]. However, heterotrophic organisms including cephalopods are subject to biochemical limitations in biosynthesis and modification of fatty acids, and assimilate the fatty acids they consume, particularly the polyunsaturated fatty acids (PUFA), in their basic form [44–48]. Fatty acids therefore have potential as dietary tracers in marine systems, providing insight into predator-prey interactions [36, 48–51]. Fatty acid analysis has proved to be a viable way to understand cephalopod diets [52, 53], and is increasingly used as a way to understand their trophic ecology [54–58].

We used fatty acid analysis and spatial analysis to investigate the feeding strategies and spatial distribution of *U*. *duvaucelii*, *U*. *edulis*, *U*. *chinensis* and *L*. *uyii* in the coastal waters of the northern South China Sea. This area is rich in tropical and subtropical biota, including various groups of phytoplankton, zooplankton, and zoobenthos, as well as pelagic and demersal fishes and invertebrates [59]. Many species, including cephalopods, are highly abundant in the shelf communities of the northern South China Sea [31, 33, 59, 60]. As fatty acids of a heterotrophic organism effectively reflect those of its diet [44–48], our study was designed to determine (1) whether the four sympatric squids adopt an opportunistic foraging strategy by feeding on similar prey items and (2) how they coexist in a coastal area where diets overlap. Our hypothesis was that there is significant dietary overlap among the squids and spatial segregation in habitat use. Our aim was to understand the potential for trophic interactions and the degree of dietary overlap among the four species, which will assist our understanding of their feeding ecology, and possibly their inclusion in the coastal ecosystem assessments.

## Materials and methods

### Ethics statement

Specimens were collected as dead squids from the small-scale trawl fishery landings, during April 2016. The specimens were analyzed in laboratory using methods that are in line with current Chinese national standards, namely Laboratory Animals—General Requirements for Animal Experiment (GB/T 35823–2018). As all material sampled in this work obtained from commercial fishermen was already dead, there was no requirement for ethical approval of sampling protocols as it did not include live organisms.

### Study area

**Fig 1:**
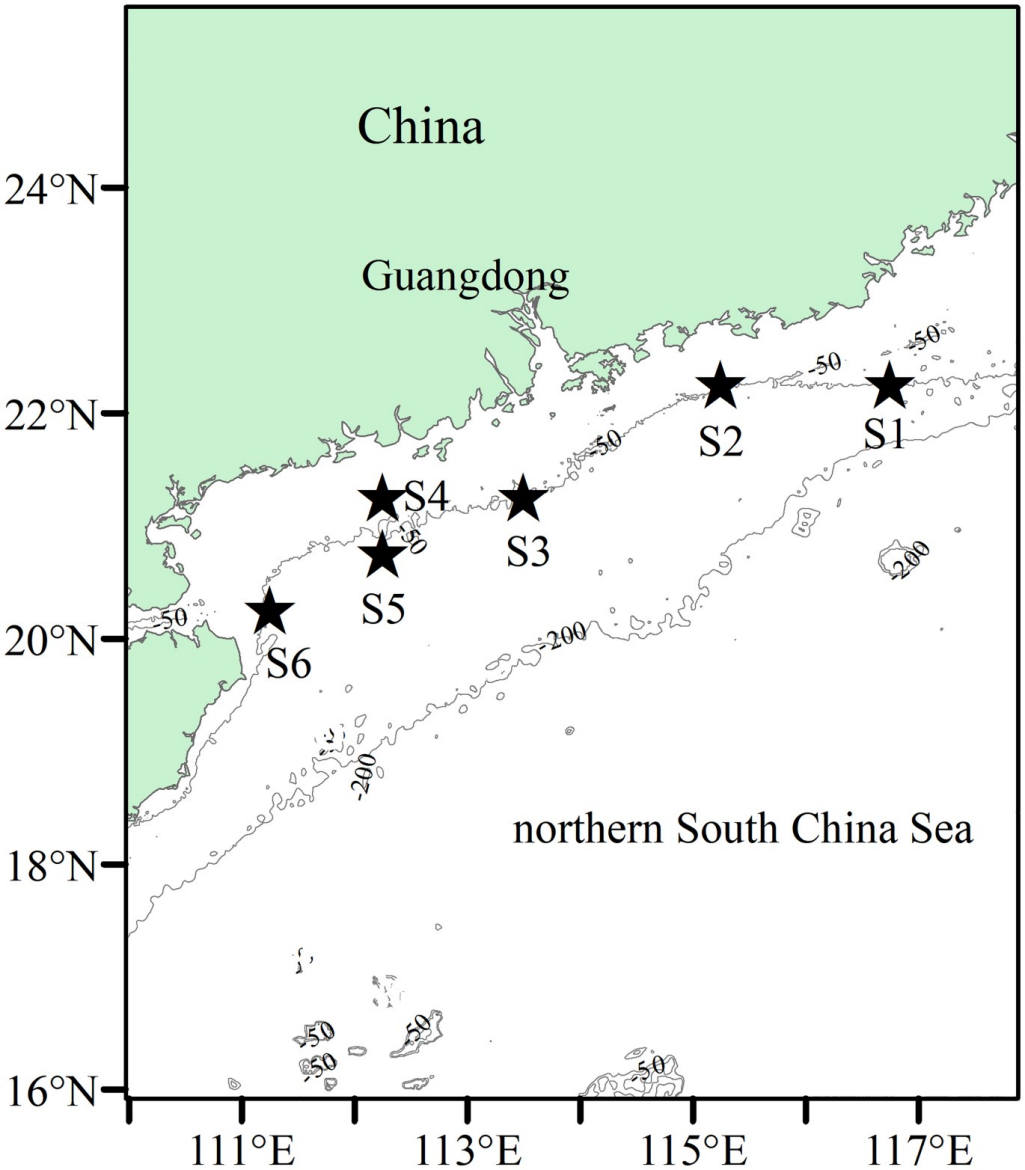
Study area and sampling stations in the northern South China Sea. Stars indicate the sampling stations. Grey lines indicate the selected isobaths of -50m and -200m.

Data collection was conducted in Guangdong coastal waters, northern South China Sea (Fig 1). This area is characterized by a broad shelf (< 200m depth), and oceanographically involves a complex circulation system that is controlled by monsoons, the Kuroshio intrusion, upwelling in summer and downwelling in winter [61–63]. The Pearl River discharges a large amount of freshwater into Guangdong coastal waters, forming an approximate plume current, which interacts with nearshore circulation due to the warm and saline South China Sea Warm Current [61, 63]. These features lead to a highly complex and dynamic ecosystem, with high nitrate concentrations and enhanced primary production [64, 65].

### Sample collection

Squid were randomly collected from the landings of a small-scale trawl fishery from April 2^nd^ to April 26^th^ 2016, at six stations (Fig 1). Similar to Philips et al. [56] and Pethybridge et al. [58], the whole squid were stored immediately at -30°C after being taken onboard. A total of 709 specimens were randomly sampled and duly labeled, including 286 *U*. *duvaucelii*, 66 *U*. *edulis*, 257 *U*. *chinensis* and 100 *L*. *uyii* (Table 1).

**Table 1:** Summary of squid specimens (n = 709) collected from the northern South China Sea, and those used in the fatty acid analyses.

Species	Sampling station	Sampled				Analyzed fatty acid samples			
		n	Mantle length (ML, mm)			n	Mantle length (ML, mm)		
			mean±sd	min	max		mean±sd	min	max
*Uroteuthis duvaucelii*	S4	13	109.2±23.3	52	158	6	116.5±15.8	92	130
	S6	273	94.2±20.9	50	165	11	98.8±21.3	75	131
	pooled	286	95.2±21.4	50	165	17	105.1±20.9	75	131
*Uroteuthis edulis*	S1	48	183.2±21.2	143	241	8	181.8±11.3	165	195
	S4	7	130.0±37.2	90	176	3	168.7±7.0	162	176
	S5	11	154.6±29.0	89	186	3	173.7±10.8	166	186
	pooled	66	165.1±36.2	89	241	14	177.2±11.3	162	195
*Uroteuthis chinensis*	S1	96	208.7±32.4	134	275	6	202.7±34.3	171	246
	S2	39	174.3±37.0	82	226	4	196.0±27.0	166	226
	S3	73	206.7±39.5	148	320	8	188.1±25.5	163	240
	S5	24	179.7±36.1	66	201	3	192.7±8.0	185	201
	S6	25	155.8±23.9	108	199	3	168.3±3.2	166	172
	pooled	257	177.3±53.6	66	320	24	191.2±25.8	163	246
*Loliolus uyii*	S4	100	69.3±5.8	55	84	7	71.9±7.3	59	79
	pooled	100	69.3±5.8	55	84	7	71.9±7.3	59	79

Sampling station corresponds to the stars in Fig 1.

In the laboratory, a subsample of 62 specimens (3 to 8 specimens of each species per sampling station) was randomly selected from the 709 specimens for fatty acid analyses (Table 1). Before defrosting, muscle tissue (~ 10.0g wet weight) from the ventral mantle of each selected specimen was obtained, and placed immediately in a drying chamber (Crhist Alpha 1-4/LDplus, Germany) to lyophilize it to a constant weight. Each dried sample was then ground into powder, and about 0.2 g of that used for fatty acid analysis. After defrosting at room temperature, measurements of dorsal mantle length (ML, in mm) were taken to the nearest 1 mm (Table 1).

### Lipid and fatty acid analyses

Fatty acid methyl esters (FAME) were analyzed for each tissue sample using a modification of the GAQSIQ [66] method. This modification was to use a mixture of chloroform and methanol 2:1 (v/v) [67] rather than diethyl ether to extract lipids [66]. The extracted lipid of each tissue sample was immediately subject to FAME analysis to avoid contamination and oxidation. This involved the addition of 4 mL 0.5mol/L KOH-MeOH to the lipid extract, incubated at 90°C for 10 minutes. Then 4 mL BF3-MeOH were added, and the solution was incubated at 90°C for 30 minutes, followed by the addition of 4 mL n-Hexane for 2 minutes incubation at a similar temperature. After adding 10 mL saturated NaCl, the solution was stratified at room temperature. Finally, the upper hexane layer was transferred to a vial, evaporated under nitrogen current.

The fatty acid profile for each sample was determined using an Agilent 7890B Gas Chromatograph coupled to a 5977A series Mass Spectrometer Detector (MSD, Agilent Technologies, Inc. USA). Methyl nonadecanoate (19:0) was used as an internal standard. Injection of samples was carried out in splitless mode, using helium as the carrier gas, and a thermal gradient from 125°C to 250°C, with an auxiliary heater at 280°C. Fatty acids were identified by comparison to relative retention times of a known standard [66] and comprise the fatty acid data for this study.

Total content of fatty acids (total FAs) was based on dry tissue weight (mg/g dry weight), and each fatty acid (FA) was reported as a percentage of the total [54–58]. The individual fatty acids were grouped into three main FA classes: saturated fatty acids (SFA), monounsaturated fatty acids (MUFA), and polyunsaturated fatty acids (PUFA). Fatty acids that accounted for <0.5% were excluded from statistical analyses.

### Statistical analysis

The fatty acid data were checked for normality (S1 Table), using a one-sample Kolmogorov-Smirnoff test [68]. The means of each FA profile, main FA class and total FAs by species and sampling station were compared using one-way analysis of variance (ANOVA) followed by Tukey’s post-hoc test if necessary [68]. The data were analyzed using the Kruskall-Wallis nonparametric test and the Games-Howell post-hoc test when the assumption of normality could not be supported [68].

Permutational multivariate analysis of variance (PERMANOVA) [69], using the Bray-Curtis similarity measure, was used to test whether the FA profiles were related to individual size or sampling station (assessed independently). The analyses were carried out for each species, as well as for the four species combined. The squids were categorized into eight size-classes (< = 80mm, 81-100mm, 101-120mm, 121-140mm, 161-180mm, 181-200mm, 201-220mm and >220mm ML), starting with the smallest individual among the four squids, for the analyses related to size of individuals. *Loliolus uyii* was not tested the influence of individual size or sampling station on the fatty acid composition since it occurred in only one size-class (<= 80 mm ML) and at a single sampling station. Canonical analysis of principal coordinates (CAP) [69] was also used to discriminate between *a priori* groupings based on size-class or sampling station, and to visualize the potential groupings from the PERMANOVA. The sample sizes for each factor level of size-class or sampling station are summarized in Table 2 and S6 Table.

**Table 2:** Sample size for each factor level of size-class and sampling station for the permutational multivariate analysis of variance (PERMANOVA) and canonical analysis of principal coordinates (CAP).

Size-class		Sampling station (see Fig 1)	
Bin groups (mm)	n	factor level	n
<80	9	S1	14
81–100	5	S2	4
101–120	6	S3	8
121–140	4	S4	15
161–180	19	S5	6
181–200	12	S6	15
201–220	4		
>220	3		

Nonmetric multidimensional scaling (nMDS) and analysis of similarities (ANOSIM), employing the Bray-Curtis similarity measure, were applied to the FA compositions by species to assess whether the species had similar feeding strategy. Morisita’s index of overlap [70], calculated using the full data set (709 specimens) from the 6 sampling stations, was used to determine whether the spatial distributions of each species were aggregated or separated in the coastal waters. The formula for the index is:

$$C_{H}=\frac{2\sum_{i}^{n}x_{ij}y_{ik}}{\sum_{i}^{n}x_{ij}^{2}+\sum_{i}^{n}y_{ik}^{2}}$$

Where C_H_ is the overlap index between species j and species k, x_ij_ is the percentage of species j at station i, y_ik_ is the percentage of species k at station i, and n is the total number of sampling stations.

All statistical analyses were conducted using OriginPro version 2015 [71] and R version 3.5.0 [72]. The multivariate analyses (PERMANOVA, CAP, nMDS, ANOSIM) and the calculation of Morisita’s index of overlap were conducted using the ‘vegan’ and ‘divo’ packages in R, respectively. Prior to the multivariate analyses, data were square-root transformed to account for variation in FA abundance. Differences were considered statistically significant when *P* < 0.05.

## Results

A total of 33 FAs were identified in the four species, with 18 having relative mean content > 0.5% of the total FAs (Table 3). These 18 FAs made up 96% of total FAs in *U*. *duvaucelii*, 95% in *U*. *edulis*, 98% in *L*. *uyii* and 97% in *U*. *chinensis*.

**Table 3:** Relative abundance of fatty acids for *Uroteuthis duvaucelii*, *Uroteuthis edulis*, *Loliolus uyii*, *Uroteuthis chinensis* in northern South China Sea.

	Species			
	*Uroteuthis duvaucelii*	*Uroteuthis edulis*	*Loliolus uyii*	*Uroteuthis chinensis*
Fatty acid (%TFA)				
14:0	2.63±3.10	2.25±2.09	2.66±0.84	2.33±1.46
16:0	19.23±2.66	18.46±2.10	19.01±3.45	19.01±5.24
**16:1n7**	0.80±0.46^ab^	0.63±0.45^a^	0.97±1.20^ab^	1.79±1.56^b^
17:0	0.71±0.08	0.70±0.14	0.84±0.10	0.74±0.22
**18:0**	7.41±2.38^a^	6.51±3.17^a^	10.3±2.4^b^	7.05±2.34^a^
18:1n9t	0.66±0.40	0.77±0.71	0.69±0.14	0.79±1.13
18:1n9c	2.51±0.47	2.24±0.47	2.79±1.37	3.59±2.27
**18:2n6t**	1.72±1.10^b^	1.40±1.33^ab^	0.44±0.46^a^	0.93±0.57^ab^
18:2n6c	0.66±0.22	0.54±0.27	0.50±0.22	0.51±0.19
18:3n6	0.69±0.45	0.55±0.52	0.57±0.15	0.60±0.21
20:0	0.60±0.28	0.52±0.36	0.36±0.13	0.44±0.17
**18:3n3**	0.97±0.52^b^	0.72±0.60^ab^	0.32±0.25^a^	0.63±0.40^ab^
20:1	1.85±0.91	2.46±1.05	2.02±0.31	2.04±0.84
20:2	0.72±0.42	0.70±0.53	0.42±0.21	0.47±0.22
20:4n6 (ARA)	2.95±1.32	3.36±1.73	3.32±1.71	3.08±1.60
**22:1n9**	0.64±0.44^b^	0.49±0.52^ab^	0.16±0.11^a^	0.38±0.22^ab^
20:5n3 (EPA)	11.15±2.04	12.74±2.38	13.01±2.40	11.55±2.98
22:6n3 (DHA)	41.96±5.45	39.39±7.78	39.22±5.6	40.70±7.48
FAs<0.5%	3.40±1.76	4.09±3.17	1.76±1.14	2.94±1.84
Main FA Classes (%TFA)				
SFA	32.59±4.05	30.70±4.97	34.25±4.78	31.12±5.98
**MUFA**	7.71±2.02^ab^	8.28±2.84^ab^	6.70±2.82^a^	9.64±3.5^b^
PUFA	59.71±4.43	61.03±6.12	59.05±4.90	59.24±7.94
Total fatty acids (mg/g dry weight)				
**TFA**	66.20±9.78^c^	59.01±3.35^b^	71.07±4.62^c^	50.71±4.54^a^

FAs <0.5% include 11:0, 12:0, 13:0, 14:1n5, 15:0, 15:1n5, 17:1n7, 21:0, 20:3n6, 22:0, 20:3n3, 23:0, 22:2n6, 24:0, 24:1n9. ARA, arachidonic acid; EPA, eicosapentaenoic acid; DHA, docosahexaenoic acid; SFA, saturated fatty acids; MUFA, monounsaturated fatty acids; PUFA, polyunsaturated fatty acids; TFA, total fatty acids. Values are mean ± standard deviation; TFA is reported as dry tissue weight (mg/g dry weight), other values are reported as percentages of TFA (% TFA). Fatty acids highlighted in bold indicate significant differences (P<0.05) among species. Superscripted letters within rows represent the results of post-hoc test, and different letters indicate significant differences in the relative content of FA between species.

### Fatty acid profiles

Significant differences in total FAs were found between species (*F* = 30.10, *P*<0.05), with *L*. *uyii* having highest total FAs, followed by *U*. *duvaucelii*, while *U*. *chinensis* had the lowest total FAs (Table 3, S2 Table). No significant differences in the relative content of the main FA classes were detected, with the exception of MUFA (*χ*^*2*^ = 8.53, *P* = 0.036), for which the highest amount was found in *U*. *chinensis* and the lowest in *L*. *uyii* (Table 3, S2 and S3 Tables).

No significant difference was detected in the relative content of 14:0 among species (*H* = 4.94, *P* = 0.18), and similar results were obtained for 16:0, 17:0, 18:1n9t, 18:1n9c, 18:2n6c, 18:3n6, 20:0, 20:1, 20:2, 20:4n6, 20:5n3 and 22:6n3 (S2 and S3 Tables). These FAs constituted 77–88% (mean±SD, 84.91±3.15) of the total FAs in *U*. *duvaucelii*, 72–92% (84.77±5.56) in *U*. *edulis*, 80–89% (84.72±3.15) in *L*. *uyii*, and 76–90% (85.35±3.25) in *U*. *chinensis*, respectively. There were significant differences in the relative content of other fatty acids (16:1n7, 18:0, 18:2n6t, 18:3n3 and 22:1n9) among species (S2 and S3 Tables); *U*. *chinensis* had the highest level of 16:1n7, *L*. *uyii* the highest level of 18:0, and *U*. *duvaucelii* the highest levels of 18:2n6t, 18:3n3 and 22:1n9 (Table 2).

### Similarity of fatty acid composition among species

**Fig 2:**
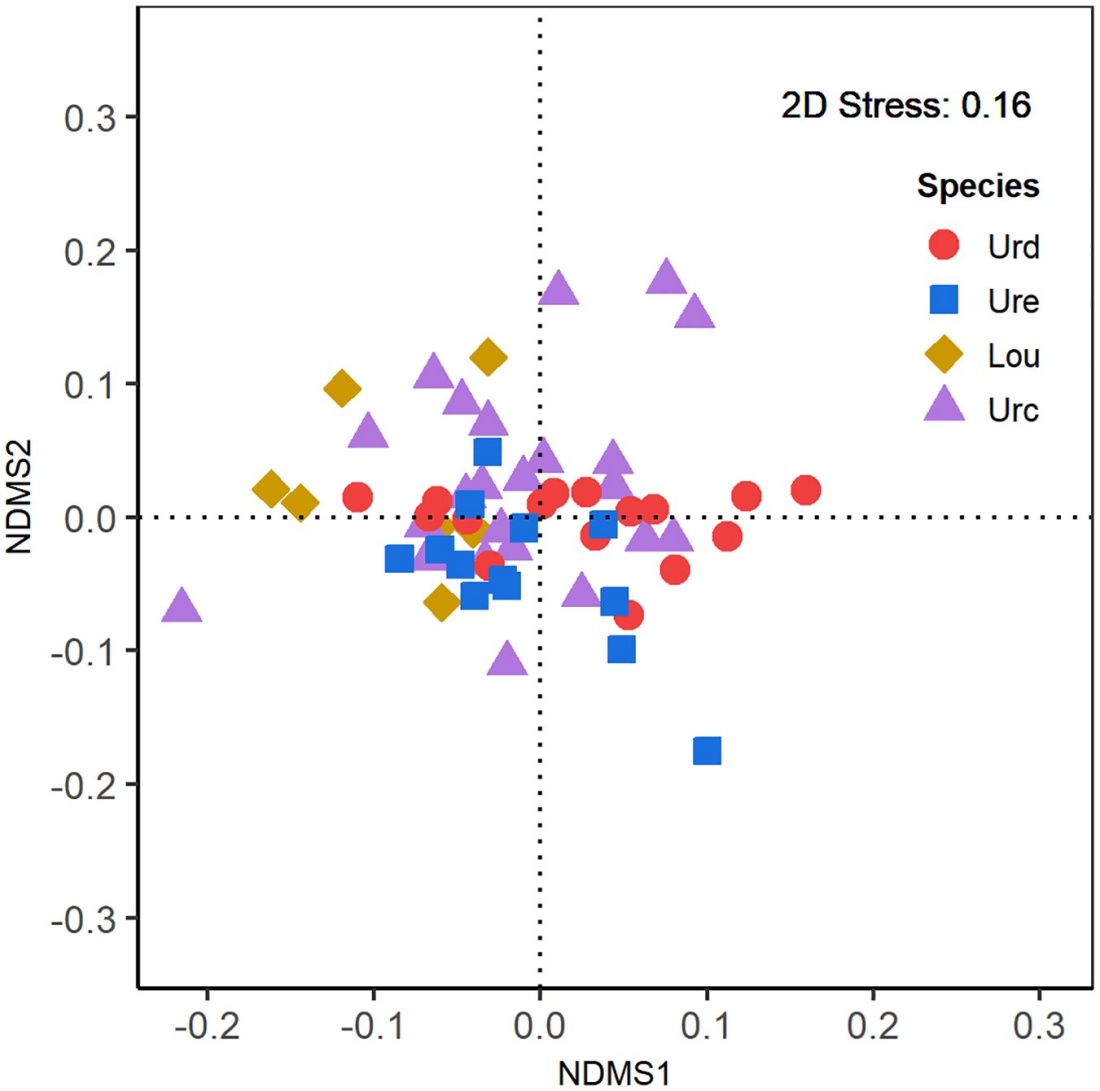
Non-metric multidimensional scaling (nMDS) ordination of fatty acid composition among *Uroteuthis duvaucelii*, *Uroteuthis edulis*, *Uroteuthis chinensis and Loliolus uyii* in the northern South China Sea. Urd, *Uroteuthis duvaucelii*; Ure, *Uroteuthis edulis*; Lou, *Loliolus uyii*; Urc, *Uroteuthis chinensis*.

The nMDS indicated considerable overlap in fatty acid profiles when the data for the four species were combined (Fig 2), and in paired species comparisons (S1 Fig). The overlap between *U*. *duvaucelii* and *L*. *uyii* and between *U*. *edulis* and *L*. *uyii* appeared to be relatively smaller than the other overlaps (S1 Fig). These findings were confirmed using ANOSIM (*R* = 0.08; “pooled” in Table 4). There was considerable similarity in the fatty acid profiles among species, except between *U*. *duvaucelii* and. *L*. *uyii* (ANOSIM *R* = 0.36) (Table 3).

**Table 4:** Results of the analysis of similarities (ANOSIM) for the fatty acid composition among *Uroteuthis duvaucelii*, *Uroteuthis edulis*, *Uroteuthis chinensis and Loliolus uyii* in the northern South China Sea.

Terms	*R*	*P*
Pooled	0.08	0.04
*U*. *duvaucelii* vs. *U*. *edulis*	0.13	0.05
*U*. *duvaucelii* vs. *L*. *uyii*	0.36	0.01
*U*. *duvaucelii* vs. *U*. *chinensis*	0.06	0.11
*U*. *edulis* vs. *L*. *uyii*	0.12	0.14
*U*. *edulis* vs. *U*. *chinensis*	0.003	0.43
*L*. *uyii* vs. *U*. *chinensis*	0.05	0.33

R ranges from -1 to 1, with values close to 0 indicating high similarity.

### Effects of individual size and sampling station on the fatty acid composition

**Fig 3:**
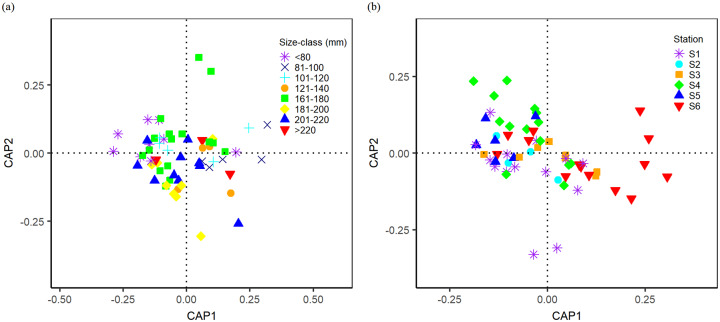
Canonical analysis of principal coordinates (CAP) based on size-class (a) and sampling station (b) of the fatty acid profiles among *Uroteuthis duvaucelii*, *Uroteuthis edulis*, *Loliolus uyii* and *Uroteuthis chinensis* in northern South China Sea. Station in (b) corresponds to the stars in Fig 1.

PERMANOVA found no effects of individual size on fatty acid composition for any of the species (*U*. *duvaucelii*, *F* = 0.66, *p* = 0.69; *U*. *edulis*, *F* = 1.64, *p* = 0.11; *U*. *chinensis*, *F* = 0.91, *p* = 0.57) and when the data were aggregated over species (*F* = 1.45, *p* = 0.07). No distinct groupings of fatty acid profiles were found when the data were grouped by size-class (CAP *p* = 0.07, Fig 3a).

There were no significant differences in the relative content of each fatty acid profile and the main FA classes (SFA, MUFA, PUFA) between sampling stations within *U*. *duvaucelii*, *U*. *edulis* and *U*. *chinensis* (S4 and S5 Tables). Similar results were obtained for the analysis of the effect of sampling station on fatty acid composition using PERMANOVA for *U*. *duvaucelii* (*F* = -0.001, *p* = 0.99), *U*. *edulis* (*F* = 1.05, *p* = 0.42), *U*. *chinensis* (*F* = 0.95, *p* = 0.54), and when the data were aggregated over species (*F* = 1.14, *p* = 0.29). Obvious overlap in fatty acid profiles among the sampling stations was observed in the CAP ordination (*p* = 0.25, Fig 3b).

### Spatial overlap

**Fig 4:**
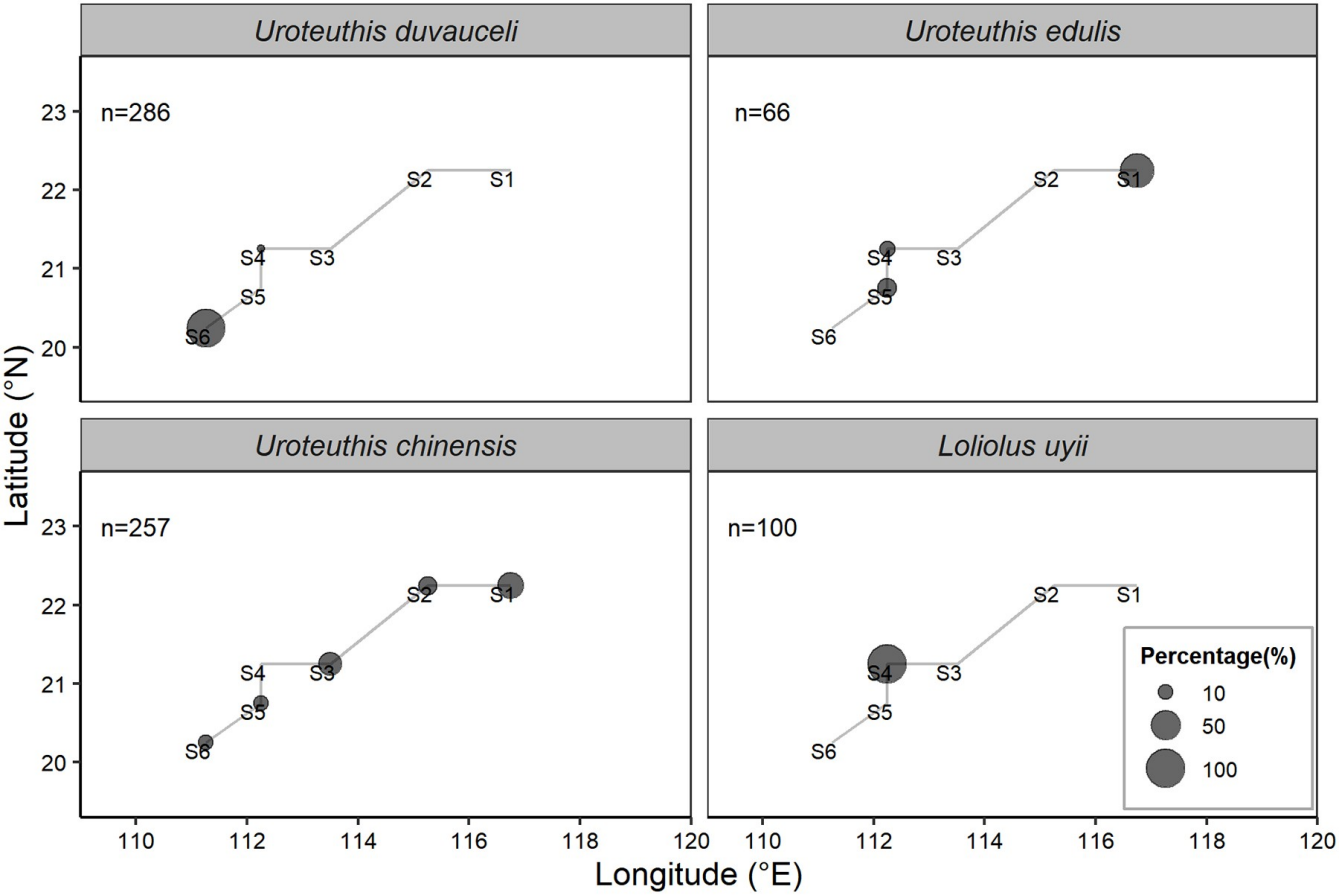
Percentage of specimens collected by sampling station for *Uroteuthis duvaucelii*, *Uroteuthis edulis*, *Loliolus uyii*, *Uroteuthis chinensis*. S1, S2,….S6 correspond to the stations in Fig 1. The size of grey circle represents the percentage of specimens collected at the station.

Spatial distribution analyses indicated that there is spatial segregation among *Uroteuthis duvaucelii*, *U*. *edulis* and *L*. *uyii* (Fig 4). *Uroteuthis duvaucelii* was found predominantly in the southwest of the study area, with *U*. *edulis* primarily in the northeast, and *L*. *uyii* at only one of center stations. A broader distribution was observed for *Uroteuthis chinensis*, which was found at the five out of the six sampling stations, with higher abundance in the northeast (Fig 4). Consequently, there appears to be considerable spatial distribution niche overlap between *U*. *chinensis* and *U*. *edulis* (Moristita’s index of 76.1%; Table 5). In contrast, lesser spatial overlap was observed between *U*. *duvaucelii* and *U*. *chinensis*, and between *U*. *edulis* and *L*. *uyii*, (Moristita’s indices of 14.6% and 12.0%, respectively). There was complete spatial segregation between *U*. *chinensis* and *L*. *uyii* (Moristita’s index = 0).

**Table 5:** Spatial niche overlap (%) among pairs of squid species—*Uroteuthis duvaucelii*, *Uroteuthis edulis*, *Loliolus uyii*, *Uroteuthis chinensis* in northern South China Sea.

Species	Urd	Ure	Urc	Lou
*Uroteuthis duvaucelii* (Urd)	-			
*Uroteuthis edulis* (Ure)	0.6%	-		
*Uroteuthis chinensis* (Urc)	14.6%	76.1%	-	
*Loliolus uyii* (Lou)	4.6%	12.0%	0	-

Percentages were calculated based on Morisita’s index.

## Discussion

The coastal waters of the northern South China Sea are characterized by high nitrate concentrations and enhanced primary production, which is responsible for the variety and abundance of tropical and subtropical biota [32, 59, 60, 64, 65]. These features could affect the feeding ecology of predators, especially species such as squids that are voracious and opportunistic predators [12, 19–20, 26]. Here, we demonstrate that the four sympatric squids, *U*. *duvaucelii*, *U*. *edulis*, *U*. *chinensis* and *L*. *uyii*, appear to be opportunistic carnivores, unselectively foraging on common prey items in the coastal waters of the northern South China Sea. There is also a clear spatial segregation among the four squids, which arises from niche differences [3–5, 73]. The spatial segregation appears to be a mechanism to reduce competition in resource use for these sympatric species in the coastal water of the northern South China Sea.

There were significant differences in the total content of fatty acid profiles among *U*. *duvaucelii*, *U*. *edulis*, *U*. *chinensis* and *L*. *uyii* probably due to variation in their lipid contents. Fatty acids form an essential and integral part of living organism’s lipids, the content of which in turn is responsible for the total amount of fatty acids [36, 48, 52]. Although squids are well documented for low lipid content (usually 2% on a wet weight basis [40, 74, 75]), there appear to be species-specific differences in the lipid content. For example, it has been reported that the lipid content of muscle tissue on a wet weight basis is around 0.8% for *Todarodes filippovae* [58] compared to 2% for *Onykia ingens* [56]. Thus, although we have not determined the lipid content for the four squid in this study, it would be expected that the difference in the total FA content is the result of different lipid content due to phylogenetic differences [43, 47].

The revealed little variation in the relative content of the main FA classes (i.e., SFA, PUFA) among the four squids is mainly contributed by the insignificant differences in the relative content of most individual FAs used as trophic markers in aquatic systems. These individual FAs include 16:0, 20:4n6, 20:5n3 and 22:6n3 [36, 76–78]. 20:5n3 indicates diatom-based food web and is identified as tracer for first-order carnivores [36, 78], 16:0 and 22:6n3 are respectively important tracers for omnivorous copepods and dinoflagellates, and are used as tracers for second-order carnivores [36, 78, 79], and 20:4n6 is a recognized indicator for benthic markers and top predators [76, 78]. Thus, the similarity in elevated levels of these FAs among the four squids implies that these species display first-order and second-order carnivore benthic feeding habit, an indication of opportunistic carnivore foraging strategy in the study area. Additional evidence can be provided by the little variation in 16:1n7 between *U*. *duvaucelii*, *U*. *edulis* and *L*. *uyii*, and the insignificant difference in 18:0 among the three *Uroteuthis* species, in which 16:1n7 and 18:0 are recognized respectively as indicators for first-order carnivores and second-order carnivores [78].

The similarity in the relative content of fatty acid profiles among the squids indicates species to prey on similar prey items, given heterotrophes generally exhibit parallel patterns of change in their FAs as they change their diets [36, 40, 46, 47, 50]. This is statistically confirmed by the multivariate analyses, in which there was substantial overlap and high similarity in the fatty acid compositions among and between pairs of species (Fig 2, S1 Fig). Although there is no definitive way to determine and quantify the prey items on which these squids fed, the high overlap of the fatty acid profiles of these species which justify sharing similar prey items, corresponds with the results of stomach contents by Islam et al. [26] who reported that *U*. *chinensis* and *U*. *duvaucelii* from the southwestern Gulf of Thailand displayed dietary similarity by feeding on three major diet groups representing crustaceans, fish and molluscs. It is worthy to note that each pair of the three *Uroteuthis* species consistently showed high similarity in fatty acid composition compared to the high dissimilarity in the fatty acid composition between *L*. *uyii* and *U*. *duvanucelii* and the significantly low levels of 18:2n6t, 18:3n3 and 22:1n9 in *L*. *uyii*, possibly suggesting phylogenetic differences in the bioaccumulation of fatty acids among these species [43, 47]. Further research, however, are needed to address the effects of phylogeny, as which is increasingly less important in higher trophic groups [78].

We also found that the fatty acid composition among the squids did not change with increasing body size. This feature suggests that these squids may not shift diet ontogenetically, but instead adopt a strategy that focuses on the amount and not quality of food as their voracious feeding habits [12, 19–20, 26]. The abundant food resources in the coastal waters of the northern South China Sea [32, 59, 60] may be a possible reason and meet their requirements with ontogeny. Islam et al. [26] also reported that *U*. *chinensis* and *U*. *duvaucelii* in the southwest of the Gulf of Thailand of different size-classes had high overlap in their food items and minimal dietary shift with ontogeny. Preying on the common prey items with increasing size may be an optimal foraging strategy for squid to maximize energy intake, enhance their growth rate and minimize predation risk [80, 81]. We also found non-significant effects of sampling station on the fatty acid composition for either species and among the four squids considered simultaneously. This observation may suggest that the squids unselectively exploit common prey items, presumably owing to the fairly stable species diversity of the northern shelf of South China Sea [33].

It is known that overlap in dietary resources between sympatric species may lead to spatial segregation, which appears to be the essential for coexistence [2, 73, 81]. The four squids exhibit the characteristics of demersal predators, evidenced by the non-significant differences and high levels in the relative content of 16:0, 20:5n3 and 20:4n6, which are indicators for demersal habitat [76, 78]. However, we found a clear spatial separation of the four squids in the coastal waters, leading to low niche overlap among them except for *U*. *chinensis* and *U*. *edulis* (Fig 3; Table 4). Regarding the spatial overlap between *U*. *chinensis* and *U*. *edulis*, we found that the former distributed broadly (five out of the six sample stations) and evenly whereas the latter was found predominantly in the northeast of the survey area. *L*. *uyii* was only found at one sampling station inside the 50m isobath (Figs 1 and 4), and appears to occupy a more brackish area as evidenced by the significantly higher level of 18:0, an indicator for brackish habitats [78]. These observations suggest that the four squids likely exploit different parts of the coastal waters. Similar patterns of spatial partitioning have been reported for the sympatric squids *I*. *argentinus*, *D*. *gahi*, and *O*. *ingens* off the Patagonian Shelf, where they exploit similar prey resources, with evidence for spatial segregation between mature *D*. *gahi* and other squids [15]. Thus, spatial segregation may be one of the coexistence mechanisms for these sympatric squids to reduce competition in resource use such as dietary sources.

## Conclusion

In conclusion, our findings indicate that the four sympatric squids *U*. *davaucelii*, *U*. *edulis*, *U*. *chinensis* and *L*. *uyii* are opportunistic carnivores, adopting a similar foraging strategy by unselectively preying on common prey items in the coastal waters of the northern South China Sea. Spatial segregation is likely a major mechanism that promotes their coexistence by reducing competition for food resources, as well as possibly buffering their trophic interactions. The abundant prey resources of the shelf waters of the northern South China Sea may allow them to adopt the similar feeding strategy and also enhance the likelihood of coexistence within the studied area. This is the first study to use fatty acid profiles to study the feeding ecology of coastal squids in the northern South China Sea. The findings advance our understanding of the feeding ecology of these sympatric squids. More importantly, our results provide a new perspective on their ecology and illustrate how fatty acids can be used to understand feeding strategy in terms of food resource use and species coexistence.

## Supporting information

S1 Table The results of the one-sample Kolmogorov-Smirnoff test for each fatty acid content among *Uroteuthis duvaucelii*, *Uroteuthis edulis*, *Loliolus uyii*, *Uroteuthis chinensis* in northern South China Sea. (DOCX)

S2 Table Results of one-way analysis of variance (ANOVA) by species for those fatty acids that meet the requirements of normality among *Uroteuthis duvaucelii*, *Uroteuthis edulis*, *Uroteuthis chinensis*, *Loliolus uyii* in northern South China Sea. (DOCX)

S3 Table Results of Kruskall-Wallis nonparametric test by species for those fatty acids that do not meet the requirements of normality among *Uroteuthis duvaucelii*, *Uroteuthis edulis*, *Uroteuthis chinensis*, *Loliolus uyii* in northern South China Sea. (DOCX)

S4 Table Results of one-way analysis of variance (ANOVA) by sampling stations for those fatty acids that meet the requirements of normality for *Uroteuthis duvaucelii*, *Uroteuthis edulis*, and *Uroteuthis chinensis* in northern South China Sea. (DOCX)

S5 Table Results of Kruskall-Wallis nonparametric test by species for those fatty acids that do not meet the requirements of normality for *Uroteuthis duvaucelii*, *Uroteuthis edulis*, and *Uroteuthis chinensis* in northern South China Sea. (DOCX)

S6 Table Sample size within each factor level of size-classes and smapling stations for permutational multivariate analysis of variance (PERMANOVA) and canonical analysis of principal coordinates (CAP) by *species*. (DOCX)

S7 Table The dataset of fatty acid profiles (%, relative content >0.5% of total FAs) determined for each squid specimen, including the sampling station and mantle length. (DOCX)

S1 Fig Non-metric multidimensional scaling (nMDS) ordination of fatty acid composition between each species pairing: Urd, *Uroteuthis duvaucelii*; Ure, *Uroteuthis edulis*; Lou, *Loliolus uyii*; Urc, *Uroteuthis chinensis*. (DOCX)
