# Supplementary material for: Fatty acid comparison of four sympatric loliginid squids in the northern South China Sea: Indication for their similar feeding strategy
Source: PLoS One. 2020 Jun 11;15(6):e0234250. doi: 10.1371/journal.pone.0234250 (PMC7289379; doi:10.1371/journal.pone.0234250)
Supplement: S1 Table — (DOCX) [file pone.0234250.s001.docx]

**S1 Table** The results of one-sample Kolmogorov-Smirnoff test for each fatty acid content among *Uroteuthis duvaucelii*, *Uroteuthis edulis*, *Loliolus uyii*, *Uroteuthis chinensis* in northern South China Sea

|  | Content | one-sample Kolmogorov-Smirnoff test |
| --- | --- | --- |
|  |  | *P* |
| Fatty acid (%TFA) | |  |
| **14:0** | 2.43±2.08 | **0** |
| 16:0 | 18.95±3.80 | 0.18 |
| **16:1n7** | 1.16±1.19 | **0.01** |
| 17:0 | 0.73±0.16 | 0.50 |
| 18:0 | 7.39±2.73 | 0.39 |
| **18:1n9t** | 0.68±0.82 | **0.002** |
| **18:1n9** | 2.90±1.60 | **0.01** |
| **18:2n6t** | 1.20±1.01 | **0.02** |
| 18:2n6 | 0.55±0.22 | 0.30 |
| **18:3n6** | 0.49±0.39 | **0.03** |
| **20:0** | 0.49±0.26 | **0.02** |
| **18:3n3** | 0.71±0.50 | **0.06** |
| 20:1 | 2.08±0.88 | 0.61 |
| 20:2 | 0.59±0.38 | 0.11 |
| 20:4n6 | 2.68±1.71 | 0.38 |
| **22:1n9** | 0.45±0.39 | **0.02** |
| 20:5n3 | 11.87±2.59 | 0.44 |
| 22:6n3 | 40.58±6.78 | 0.21 |
| Main FA Classes (%TFA) | |  |
| SFA | 31.78±5.16 | 0.66 |
| **MUFA** | 8.47±3.04 | **0.02** |
| PUFA | 59.75±6.31 | 0.33 |
| Total fatty acids (mg/g dry weight) | |  |
| TFA | 59.13±9.77 | 0.77 |

Values are mean ± standard deviation, estimated for the each fatty acid without considering species. SFA, saturated fatty acids; MUFA, monounsaturated fatty acids; PUFA, polyunsaturated fatty acids; TFA, total fatty acids. Values are mean ± standard deviation; TFA is reported as dry tissue weight (mg/g dry weight), other values are reported as percentages of TFA (% TFA). Fatty acid highlighted in bold indicates that the data do not meet the requirements of normality.
