# Supplementary material for: Fatty acid comparison of four sympatric loliginid squids in the northern South China Sea: Indication for their similar feeding strategy
Source: PLoS One. 2020 Jun 11;15(6):e0234250. doi: 10.1371/journal.pone.0234250 (PMC7289379; doi:10.1371/journal.pone.0234250)
Supplement: S2 Table — (DOCX) [file pone.0234250.s002.docx]

**S2 Table** Results of one-way analysis of variance (ANOVA) by species for those fatty acids that meet the requirements of normality among *Uroteuthis duvaucelii*, *Uroteuthis edulis*, *Uroteuthis chinensis,* *Loliolus uyii* in northern South China Sea

| Fatty acid | F | P |
| --- | --- | --- |
| 16:0 | 0.11 | 0.96 |
| 17:0 | 1.49 | 0.23 |
| **18:0** | 3.69 | 0.02 |
| 18:2n6c | 1.74 | 0.17 |
| 20:1 | 1.28 | 0.29 |
| 20:2 | 2.39 | 0.08 |
| 20:4n6 | 1.78 | 0.15 |
| 20:5n3 | 1.59 | 0.20 |
| 22:6n3 | 0.46 | 0.71 |
| SFA | 1.01 | 0.39 |
| PUFA | 0.26 | 0.85 |
| **TFA** | 30.10 | 0 |

SFA, saturated fatty acids; PUFA, polyunsaturated fatty acids; TFA, total fatty acids. Fatty acid highlighted in bold indicates significant differences (*P*<0.05) among species.
