# Supplementary material for: Fatty acid comparison of four sympatric loliginid squids in the northern South China Sea: Indication for their similar feeding strategy
Source: PLoS One. 2020 Jun 11;15(6):e0234250. doi: 10.1371/journal.pone.0234250 (PMC7289379; doi:10.1371/journal.pone.0234250)
Supplement: S3 Table — (DOCX) [file pone.0234250.s003.docx]

**S3 Table** Results of the Kruskall-Wallis nonparametric test by species for those fatty acids that do not meet the requirements of normality among *Uroteuthis duvaucelii*, *Uroteuthis edulis*, *Uroteuthis chinensis*, *Loliolus uyii* in northern South China Sea

| Fatty acid | H | P |
| --- | --- | --- |
| 14:0 | 4.94 | 0.18 |
| **16:1n7** | 9.10 | 0.03 |
| 18:1n9t | 6.08 | 0.11 |
| 18:1n9c | 7.22 | 0.07 |
| **18:2n6t** | 14.50 | 0.002 |
| 18:3n6 | 3.73 | 0.23 |
| 20:0 | 4.71 | 0.19 |
| **18:3n3** | 11.99 | 0.01 |
| **22:1n9** | 13.79 | 0.003 |
| **MUFA** | 8.53 | 0.04 |

MUFA, monounsaturated fatty acids. Fatty acid highlighted in bold indicates significant differences (*P*<0.05) among species.
