# Supplementary material for: Fatty acid comparison of four sympatric loliginid squids in the northern South China Sea: Indication for their similar feeding strategy
Source: PLoS One. 2020 Jun 11;15(6):e0234250. doi: 10.1371/journal.pone.0234250 (PMC7289379; doi:10.1371/journal.pone.0234250)
Supplement: S4 Table — (DOCX) [file pone.0234250.s004.docx]

**S4 Table** Results of one-way analysis of variance (ANOVA) by sampling station for those fatty acids that meet the requirements of normality for *Uroteuthis duvaucelii*, *Uroteuthis edulis*, *and* *Uroteuthis chinensis* in northern South China Sea

| Fatty acid | Species |  |  |  |  |  |
| --- | --- | --- | --- | --- | --- | --- |
|  | *Uroteuthis duvauceli* | | *Uroteuthis edulis* | | *Uroteuthis chinensis* | |
|  | F | P | F | P | F | P |
| 16.0 | 0.78 | 0.39 | 0.27 | 0.77 | 0.79 | 0.55 |
| 17.0 | 0.09 | 0.76 | 0.15 | 0.87 | 2.90 | 0.05 |
| 18.0 | 0.01 | 0.92 | 3.94 | 0.05 | 0.57 | 0.69 |
| 18.2n6c | 0.00 | 0.98 | 0.88 | 0.44 | 2.92 | 0.05 |
| 20.1 | 0.05 | 0.83 | 0.04 | 0.96 | 1.01 | 0.42 |
| 20.2 | 0.09 | 0.76 | 0.94 | 0.42 | 2.57 | 0.07 |
| 20.4n6 | 0.40 | 0.54 | 0.17 | 0.85 | 1.16 | 0.36 |
| 20.5n3 | 1.11 | 0.31 | 2.36 | 0.14 | 0.16 | 0.96 |
| 22.6n3 | 0.35 | 0.56 | 1.41 | 0.28 | 2.37 | 0.09 |
| SFA | 0.00 | 0.97 | 5.49 | 0.02 | 2.86 | 0.05 |
| PUFA | 0.02 | 0.90 | 2.94 | 0.09 | 2.47 | 0.08 |

SFA, saturated fatty acids; PUFA, polyunsaturated fatty acids
