# Supplementary material for: Fatty acid comparison of four sympatric loliginid squids in the northern South China Sea: Indication for their similar feeding strategy
Source: PLoS One. 2020 Jun 11;15(6):e0234250. doi: 10.1371/journal.pone.0234250 (PMC7289379; doi:10.1371/journal.pone.0234250)
Supplement: S5 Table — (DOCX) [file pone.0234250.s005.docx]

**S5 Table** Results of the Kruskall-Wallis nonparametric test by sampling station for those fatty acids that do not meet the requirements of normality for *Uroteuthis duvaucelii*, *Uroteuthis edulis*, *and* *Uroteuthis chinensis* in northern South China Sea

| Fatty acid | Species |  |  |  |  |  |
| --- | --- | --- | --- | --- | --- | --- |
|  | *Uroteuthis duvauceli* | | *Uroteuthis edulis* | | *Uroteuthis chinensis* | |
|  | H | P | H | P | H | P |
| C14.0 | 0.16 | 0.69 | 5.25 | 0.07 | 7.55 | 0.11 |
| C16.1n7 | 1.45 | 0.23 | 0.73 | 0.69 | 4.57 | 0.33 |
| C18.1n9t | 0.49 | 0.48 | 3.43 | 0.18 | 2.16 | 0.71 |
| C18.1n9c | 0.09 | 0.76 | 0.49 | 0.78 | 2.60 | 0.63 |
| C18.2n6t | 0.09 | 0.76 | 2.45 | 0.29 | 11.12 | 0.03* |
| C18.3n6 | 0.09 | 0.76 | 1.88 | 0.39 | 8.97 | 0.06 |
| C20.0 | 0.16 | 0.69 | 1.68 | 0.43 | 5.97 | 0.20 |
| C18.3n3 | 0.16 | 0.69 | 1.88 | 0.39 | 7.79 | 0.10 |
| C22.1n9 | 0.09 | 0.76 | 2.19 | 0.33 | 7.50 | 0.11 |
| MUFA | 0.01 | 0.92 | 1.30 | 0.52 | 2.21 | 0.70 |

MUFA, monounsaturated fatty acids. *P* value with super character “*” indicates significant differences (*P*<0.05).
