# Supplementary material for: Fatty acid comparison of four sympatric loliginid squids in the northern South China Sea: Indication for their similar feeding strategy
Source: PLoS One. 2020 Jun 11;15(6):e0234250. doi: 10.1371/journal.pone.0234250 (PMC7289379; doi:10.1371/journal.pone.0234250)
Supplement: S6 Table — (DOCX) [file pone.0234250.s006.docx]

**S6 Table** Sample size within each factor level of size-class and sampling station for permutational multivariate analysis of variance (PERMANOVA) and canonical analysis of principal coordinates (CAP) by species.

| Size-class |  | Sampling station |  |
| --- | --- | --- | --- |
| Bin groups (mm) | n | factor level | n |
| *Uroteuthis duvauceli* |  |  |  |
| <80 | 2 | S4 | 5 |
| 81-100 | 5 | S6 | 12 |
| 101-120 | 6 |  |  |
| 121-140 | 4 |  |  |
| *Uroteuthis edulis* |  |  |  |
| 161-180 | 8 | S1 | 8 |
| 181-200 | 6 | S4 | 3 |
|  |  | S5 | 4 |
| *Uroteuthis chinensis* |  |  |  |
| 161-180 | 11 | S1 | 6 |
| 181-200 | 6 | S2 | 4 |
| 201-220 | 4 | S3 | 8 |
| >220 | 3 | S5 | 3 |
|  |  | S6 | 3 |
| *Loliolus uyii* |  |  |  |
| <80 | 7 | S4 | 7 |

S1, S2,….S6 correspond to the stations in Fig. 1.
