# Supplementary material for: Fatty acid comparison of four sympatric loliginid squids in the northern South China Sea: Indication for their similar feeding strategy
Source: PLoS One. 2020 Jun 11;15(6):e0234250. doi: 10.1371/journal.pone.0234250 (PMC7289379; doi:10.1371/journal.pone.0234250)
Supplement: S7 Table — (DOCX) [file pone.0234250.s007.docx]

**S7 Table** The dataset of fatty acid profiles (%, relative content >0.5% of total FAs) determined for each squid specimen, including the sampling station and mantle length. Stn, sampling station corresponding to the stations in Fig.1.

| Species | Stn | ML(mm) | C14.0 | C16.0 | C16.1n7 | C17.0 | C18.0 | C18.1n9t | C18.1n9c | C18.2n6t | C18.2n6c | C18.3n6 | C20.0 | C18.3n3 | C20.1 | C20.2 | C20.4n6 | C22.1n9 | C20.5n3 | C22.6n3 |
| --- | --- | --- | --- | --- | --- | --- | --- | --- | --- | --- | --- | --- | --- | --- | --- | --- | --- | --- | --- | --- |
| Loliolus uyii | S4 | 59 | 3.5855 | 11.6548 | 1.1796 | 0.8430 | 7.8961 | 0.0659 | 2.8081 | 0.0669 | 0.6002 | 0.0383 | 0.2200 | 0.2099 | 2.3748 | 0.6118 | 2.0278 | 0.0550 | 17.0083 | 47.8528 |
| Loliolus uyii | S4 | 66 | 2.0800 | 21.2115 | 0.2491 | 0.9666 | 10.2000 | 0.4000 | 1.8567 | 1.0789 | 0.5294 | 0.3594 | 0.4915 | 0.4293 | 2.2826 | 0.7252 | 6.2029 | 0.2995 | 12.5223 | 30.3758 |
| Loliolus uyii | S4 | 72 | 2.2674 | 19.2647 | 0.5302 | 0.7035 | 9.4714 | 0.3452 | 2.3928 | 1.0003 | 0.5619 | 0.3449 | 0.4898 | 0.4486 | 1.6502 | 0.5393 | 4.9959 | 0.2727 | 11.7224 | 37.1870 |
| Loliolus uyii | S4 | 72 | 2.1220 | 18.9858 | 0.3221 | 0.8756 | 11.7727 | 0.0881 | 2.4003 | 0.0434 | 0.2533 | 0.0200 | 0.3074 | 0.0396 | 2.0597 | 0.2404 | 2.5661 | 0.0509 | 15.0825 | 42.2700 |
| Loliolus uyii | S4 | 76 | 2.2885 | 22.1852 | 0.3510 | 0.8556 | 14.8026 | 0.0922 | 1.9682 | 0.0574 | 0.2401 | 0.0243 | 0.1825 | 0.0655 | 1.7155 | 0.2087 | 2.1500 | 0.0401 | 10.3795 | 41.9041 |
| Loliolus uyii | S4 | 79 | 2.1640 | 20.4559 | 0.5332 | 0.7198 | 10.0218 | 0.2440 | 2.3003 | 0.6504 | 0.4311 | 0.2395 | 0.4184 | 0.3324 | 1.7572 | 0.3943 | 4.6000 | 0.1944 | 10.7607 | 39.5550 |
| Loliolus uyii | S4 | 79 | 4.1374 | 19.3070 | 3.5945 | 0.9171 | 7.9479 | 0.0794 | 5.8156 | 0.2135 | 0.8828 | 0.1305 | 0.4308 | 0.7433 | 2.3134 | 0.2131 | 2.0929 | 0.1881 | 13.6082 | 35.4108 |
| Uroteuthis chinensis | S3 | 163 | 0.5693 | 19.3073 | 0.9086 | 0.7891 | 6.7650 | 0.9730 | 1.8512 | 2.2242 | 0.5770 | 0.8911 | 0.6732 | 1.0166 | 2.2995 | 0.9739 | 2.1282 | 0.8975 | 12.1838 | 40.6010 |
| Uroteuthis chinensis | S2 | 166 | 2.0211 | 18.0261 | 1.4146 | 0.8243 | 6.4153 | 0.2513 | 3.6535 | 0.6580 | 0.6092 | 0.2739 | 0.3877 | 0.4595 | 1.0645 | 0.3654 | 3.4499 | 0.2219 | 9.9797 | 45.0652 |
| Uroteuthis chinensis | S6 | 166 | 4.0268 | 30.9388 | 3.7249 | 1.1140 | 9.0741 | 0.4148 | 5.3985 | 1.2396 | 0.9152 | 0.5750 | 0.6146 | 1.1333 | 2.5048 | 0.6271 | 1.5939 | 0.6166 | 8.3286 | 23.6093 |
| Uroteuthis chinensis | S6 | 167 | 1.4823 | 19.3432 | 0.3434 | 0.6069 | 5.8306 | 0.8971 | 1.9379 | 0.6620 | 0.4096 | 0.2318 | 0.2484 | 0.4227 | 2.7525 | 0.4700 | 2.3604 | 0.2044 | 14.0877 | 44.9293 |
| Uroteuthis chinensis | S1 | 171 | 1.6149 | 20.0028 | 1.1672 | 0.7463 | 6.4396 | 0.1723 | 4.5877 | 0.4556 | 0.4008 | 0.2038 | 0.2462 | 0.3447 | 1.3967 | 0.2660 | 2.3262 | 0.2330 | 4.7294 | 53.1317 |
| Uroteuthis chinensis | S1 | 172 | 3.0319 | 23.7553 | 3.7362 | 0.6491 | 7.6579 | 0.1508 | 3.8045 | 0.3178 | 0.5113 | 0.1919 | 0.2726 | 0.4243 | 1.2801 | 0.2392 | 1.7889 | 0.2090 | 10.3381 | 40.1679 |
| Uroteuthis chinensis | S3 | 172 | 1.3792 | 18.0450 | 0.1006 | 0.5687 | 4.5457 | 0.4211 | 1.1873 | 1.2917 | 0.4714 | 0.4220 | 0.4025 | 0.4972 | 3.7960 | 0.5644 | 5.9479 | 0.3412 | 17.6013 | 34.9797 |
| Uroteuthis chinensis | S3 | 172 | 1.8907 | 22.1324 | 1.1373 | 0.9382 | 8.7359 | 0.3540 | 3.3153 | 0.9264 | 0.5614 | 0.3622 | 0.4544 | 0.4843 | 1.5911 | 0.4816 | 4.7102 | 0.3017 | 9.1756 | 36.1386 |
| Uroteuthis chinensis | S6 | 172 | 7.4481 | 20.7897 | 5.9870 | 1.4615 | 11.0100 | 0.3585 | 6.6540 | 0.8592 | 0.9688 | 0.4956 | 0.8855 | 1.0367 | 2.2537 | 0.5104 | 1.9568 | 0.5261 | 9.3945 | 23.5556 |
| Uroteuthis chinensis | S3 | 176 | 2.0556 | 13.1128 | 1.8720 | 0.8509 | 9.8374 | 0.3883 | 4.2082 | 1.2353 | 0.4453 | 0.5229 | 0.4770 | 0.6587 | 2.4024 | 0.5883 | 3.4923 | 0.5297 | 12.1193 | 42.4329 |
| Uroteuthis chinensis | S3 | 177 | 1.2525 | 21.5646 | 1.6143 | 0.7050 | 7.0340 | 0.5400 | 3.9507 | 1.8107 | 0.5744 | 0.7374 | 0.6274 | 0.8777 | 2.2104 | 0.7838 | 2.0040 | 0.7267 | 10.2858 | 38.9374 |
| Uroteuthis chinensis | S2 | 182 | 1.6607 | 16.9860 | 0.2315 | 0.7324 | 8.3101 | 0.2651 | 1.7973 | 0.7380 | 0.4705 | 0.2476 | 0.3393 | 0.3061 | 1.4763 | 0.4000 | 4.8011 | 0.1950 | 9.8557 | 45.3301 |
| Uroteuthis chinensis | S5 | 185 | 1.8468 | 18.9728 | 0.6446 | 0.7472 | 7.4702 | 0.2007 | 2.6809 | 0.5264 | 0.3927 | 0.1931 | 0.2891 | 0.2787 | 1.5018 | 0.2969 | 4.0746 | 0.1608 | 9.1584 | 45.1521 |
| Uroteuthis chinensis | S1 | 190 | 3.0112 | 21.9343 | 2.0739 | 0.5192 | 0.1770 | 5.6494 | 0.4198 | 0.3274 | 0.1937 | 0.3027 | 0.3573 | 2.0599 | 0.1324 | 0.0921 | 0.1864 | 0.1362 | 10.1389 | 39.3803 |
| Uroteuthis chinensis | S5 | 192 | 1.4509 | 18.8100 | 0.7392 | 0.7231 | 6.5559 | 0.6692 | 2.7339 | 0.6316 | 0.3004 | 0.3153 | 0.2777 | 0.4028 | 2.3191 | 0.4036 | 2.7358 | 0.3291 | 12.4042 | 46.4041 |
| Uroteuthis chinensis | S1 | 192 | 2.2596 | 0.5634 | 0.6943 | 0.8394 | 11.9781 | 0.2729 | 3.0600 | 0.8568 | 0.3866 | 0.3401 | 0.3985 | 0.3974 | 2.0796 | 0.4437 | 4.0361 | 0.3447 | 11.4167 | 57.9394 |
| Uroteuthis chinensis | S3 | 198 | 0.9580 | 19.2346 | 0.5815 | 0.6489 | 6.8972 | 1.4753 | 2.4944 | 0.6704 | 0.2729 | 0.2782 | 0.2491 | 0.3405 | 2.6656 | 0.4151 | 2.0164 | 0.3123 | 12.8786 | 46.4416 |
| Uroteuthis chinensis | S5 | 201 | 3.1190 | 17.6322 | 3.6192 | 0.5405 | 5.6537 | 0.1714 | 6.1058 | 0.2211 | 0.5300 | 0.1882 | 0.3785 | 0.3331 | 1.0411 | 0.1939 | 2.6112 | 0.1313 | 15.3171 | 39.2357 |
| Uroteuthis chinensis | S3 | 207 | 1.3291 | 24.2177 | 1.1305 | 0.8380 | 8.8499 | 0.3791 | 3.4396 | 1.0885 | 0.4281 | 0.4458 | 0.4085 | 0.5573 | 1.5725 | 0.4856 | 2.1439 | 0.4579 | 9.0570 | 40.6612 |
| Uroteuthis chinensis | S2 | 210 | 4.8362 | 15.1649 | 2.9021 | 0.4304 | 5.6141 | 0.4104 | 11.5961 | 0.3069 | 0.6684 | 0.1989 | 0.5874 | 0.4607 | 3.2056 | 0.2166 | 0.5392 | 0.2766 | 14.8472 | 36.4908 |
| Uroteuthis chinensis | S2 | 226 | 2.7148 | 20.7328 | 2.0054 | 0.5169 | 5.2771 | 1.5102 | 3.9912 | 1.0219 | 0.4149 | 0.4824 | 0.4745 | 0.5688 | 3.1540 | 0.5479 | 1.0472 | 0.4923 | 14.1025 | 38.7302 |
| Uroteuthis chinensis | S3 | 240 | 2.3957 | 17.0214 | 0.2998 | 0.6992 | 6.7302 | 0.5988 | 2.1075 | 1.8074 | 0.8055 | 0.6087 | 0.6254 | 0.7311 | 1.5782 | 0.8036 | 6.5839 | 0.4497 | 8.9838 | 39.7493 |
| Uroteuthis chinensis | S1 | 245 | 2.1206 | 20.8260 | 4.7780 | 0.5837 | 6.6147 | 0.5179 | 3.3584 | 0.4757 | 0.3811 | 0.3570 | 0.2373 | 0.4235 | 1.6388 | 0.3257 | 2.7219 | 0.2758 | 16.1936 | 36.5314 |
| Uroteuthis chinensis | S1 | 246 | 1.4427 | 17.1333 | 1.1649 | 0.6945 | 5.6598 | 1.9215 | 1.8398 | 2.0533 | 0.5191 | 0.8499 | 0.6267 | 0.9710 | 3.0913 | 0.9003 | 1.3879 | 0.8520 | 14.5307 | 41.1107 |
| Uroteuthis duvauceli | S6 | 75 | 1.5784 | 20.1185 | 1.3812 | 0.7178 | 5.9729 | 0.5743 | 2.7036 | 1.9502 | 0.9210 | 0.7959 | 0.6492 | 1.7078 | 1.5085 | 0.8125 | 0.8708 | 0.7756 | 10.1528 | 43.1510 |
| Uroteuthis duvauceli | S6 | 75 | 2.3595 | 21.2799 | 0.2499 | 0.6690 | 7.7723 | 0.2319 | 2.6476 | 0.6619 | 0.3375 | 0.2263 | 0.3391 | 0.3078 | 1.2431 | 0.2903 | 2.4507 | 0.1822 | 10.5836 | 45.3388 |
| Uroteuthis duvauceli | S6 | 81 | 8.0559 | 15.7572 | 1.2396 | 0.7761 | 6.6204 | 0.8706 | 1.4769 | 2.9136 | 0.7899 | 1.1940 | 0.9383 | 1.6178 | 1.5165 | 1.1862 | 0.9192 | 1.1613 | 8.6714 | 39.5179 |
| Uroteuthis duvauceli | S6 | 82 | 1.1779 | 18.4571 | 0.8100 | 0.7314 | 6.8204 | 0.2761 | 3.1871 | 0.9065 | 0.7566 | 0.3851 | 0.3939 | 0.9180 | 1.1716 | 0.4280 | 0.8156 | 0.3777 | 9.8390 | 50.0597 |
| Uroteuthis duvauceli | S6 | 84 | 0.1335 | 16.9795 | 1.0437 | 0.7493 | 6.4371 | 0.7192 | 2.2822 | 2.4320 | 0.8046 | 0.9878 | 0.7686 | 1.3732 | 1.5907 | 0.9893 | 0.9732 | 0.9577 | 9.6292 | 46.1713 |
| Uroteuthis duvauceli | S6 | 89 | 1.1502 | 20.1238 | 1.1709 | 0.6596 | 7.1039 | 1.0802 | 2.5406 | 1.6120 | 0.5825 | 0.6666 | 0.5257 | 0.9189 | 3.1891 | 0.6997 | 0.8277 | 0.6687 | 13.9887 | 39.8122 |
| Uroteuthis duvauceli | S4 | 92 | 1.0898 | 20.8522 | 0.5793 | 0.6866 | 7.5702 | 0.3182 | 3.0501 | 1.0800 | 0.4938 | 0.4341 | 0.3934 | 0.7001 | 1.6031 | 0.4710 | 0.6299 | 0.4422 | 10.3802 | 47.3179 |
| Uroteuthis duvauceli | S4 | 102 | 12.6072 | 13.3680 | 1.4677 | 0.8340 | 5.7426 | 1.5297 | 2.0619 | 4.6058 | 1.0647 | 1.8452 | 1.3422 | 2.1070 | 3.2261 | 1.8029 | 0.6035 | 1.7955 | 10.2424 | 26.6878 |
| Uroteuthis duvauceli | S6 | 112 | 2.9818 | 17.9124 | 1.1222 | 0.7881 | 7.1818 | 1.1166 | 2.2115 | 3.3745 | 0.8508 | 1.3251 | 0.9875 | 1.5508 | 2.6502 | 1.3221 | 0.8981 | 1.2955 | 10.9511 | 36.3612 |
| Uroteuthis duvauceli | S6 | 118 | 0.9157 | 21.5048 | 0.5026 | 0.7380 | 7.0601 | 0.6101 | 2.2177 | 1.6464 | 0.4484 | 0.7052 | 0.4112 | 0.6558 | 2.8612 | 0.5414 | 0.6566 | 0.5368 | 12.2761 | 43.6402 |
| Uroteuthis duvauceli | S4 | 120 | 1.9744 | 22.8986 | 0.2709 | 0.7225 | 14.5149 | 0.1640 | 2.7601 | 0.5522 | 0.3121 | 0.2060 | 0.3625 | 0.2987 | 1.0833 | 0.2513 | 0.9155 | 0.2091 | 9.2781 | 42.2996 |
| Uroteuthis duvauceli | S6 | 120 | 2.1877 | 19.3802 | 0.4922 | 0.5342 | 9.3176 | 0.3280 | 2.7159 | 0.9515 | 0.4541 | 0.3281 | 0.4717 | 0.5356 | 1.1497 | 0.3992 | 2.9769 | 0.2640 | 10.1690 | 43.9878 |
| Uroteuthis duvauceli | S6 | 120 | 2.2956 | 21.0299 | 0.3135 | 0.7911 | 11.2496 | 0.2794 | 3.2564 | 0.8069 | 0.5865 | 0.2886 | 0.4682 | 0.5072 | 1.1830 | 0.3876 | 2.6248 | 0.2238 | 9.5745 | 41.1655 |
| Uroteuthis duvauceli | S4 | 127 | 1.4548 | 18.2899 | 0.3980 | 0.7258 | 6.2187 | 0.6219 | 2.9052 | 0.8819 | 0.6550 | 0.3117 | 0.3502 | 0.6534 | 0.2575 | 0.4147 | 2.5114 | 0.2595 | 12.6978 | 47.4446 |
| Uroteuthis duvauceli | S4 | 128 | 2.1673 | 18.9869 | 0.2408 | 0.6141 | 4.3888 | 0.8400 | 2.0442 | 2.1597 | 0.9251 | 0.8463 | 0.7993 | 1.0388 | 1.8215 | 0.9794 | 5.6366 | 0.6422 | 11.6585 | 37.2523 |
| Uroteuthis duvauceli | S4 | 130 | 0.5069 | 16.3326 | 0.6644 | 0.6150 | 5.5172 | 1.2117 | 1.9969 | 1.5139 | 0.4918 | 0.6268 | 0.4736 | 0.7746 | 3.5157 | 0.6699 | 0.6721 | 0.6277 | 16.8375 | 44.2486 |
| Uroteuthis duvauceli | S6 | 131 | 1.9969 | 23.7029 | 1.5821 | 0.6752 | 6.5276 | 0.5081 | 2.5310 | 1.2356 | 0.6714 | 0.5424 | 0.4628 | 0.8257 | 1.9002 | 0.6186 | 1.3595 | 0.5401 | 12.5939 | 38.8512 |
| Uroteuthis edulis | S4 | 162 | 1.9396 | 18.5000 | 0.1567 | 0.7548 | 10.3636 | 0.3260 | 2.0125 | 0.8660 | 0.3310 | 0.2880 | 0.3984 | 0.3479 | 2.2677 | 0.3555 | 3.9564 | 0.2350 | 12.9123 | 39.2588 |
| Uroteuthis edulis | S1 | 165 | 1.5475 | 17.9711 | 0.3236 | 0.8565 | 6.2739 | 0.7184 | 2.1873 | 2.1448 | 0.8071 | 0.7178 | 0.6951 | 0.8576 | 2.9989 | 0.8968 | 6.5488 | 0.5523 | 11.5460 | 35.4693 |
| Uroteuthis edulis | S1 | 166 | 0.8191 | 18.1628 | 1.2054 | 0.8572 | 8.1481 | 0.6251 | 2.4102 | 1.6408 | 0.6116 | 0.8132 | 0.6495 | 0.9731 | 1.5727 | 0.8820 | 3.0528 | 0.7976 | 11.5413 | 41.8871 |
| Uroteuthis edulis | S5 | 166 | 1.5876 | 17.2792 | 0.1326 | 0.7751 | 12.6343 | 0.2488 | 1.9860 | 0.6758 | 0.2924 | 0.2311 | 0.3725 | 0.2858 | 1.7003 | 0.2989 | 3.5947 | 0.1863 | 12.2695 | 41.1527 |
| Uroteuthis edulis | S4 | 168 | 2.9846 | 20.6260 | 0.6505 | 0.5579 | 9.1571 | 0.3815 | 2.6474 | 1.1094 | 0.5006 | 0.3880 | 0.4875 | 0.5130 | 1.1338 | 0.4651 | 3.9075 | 0.3048 | 8.3049 | 41.7600 |
| Uroteuthis edulis | S5 | 169 | 2.5680 | 23.8040 | 0.7467 | 0.9108 | 8.6069 | 0.3641 | 3.1294 | 1.0339 | 0.6121 | 0.3742 | 0.4682 | 0.5033 | 2.4040 | 0.5599 | 5.6138 | 0.3025 | 13.7939 | 27.2100 |
| Uroteuthis edulis | S4 | 176 | 9.2286 | 15.8199 | 1.7046 | 0.7171 | 7.2600 | 1.8776 | 1.9496 | 5.6423 | 1.2548 | 2.2148 | 1.6107 | 2.4550 | 4.1234 | 2.3784 | 1.0779 | 2.1890 | 10.5378 | 19.5073 |
| Uroteuthis edulis | S1 | 179 | 1.2117 | 16.4502 | 0.7641 | 0.5614 | 5.5193 | 0.7837 | 2.4199 | 0.5853 | 0.3027 | 0.2533 | 0.2096 | 0.3292 | 3.0155 | 0.6555 | 2.9389 | 0.2616 | 16.0559 | 46.2736 |
| Uroteuthis edulis | S1 | 183 | 1.6139 | 20.3600 | 0.1838 | 0.4775 | 3.9440 | 0.3601 | 1.4043 | 1.0949 | 0.4343 | 0.3692 | 0.3637 | 0.4416 | 2.3822 | 0.4831 | 3.9022 | 0.2932 | 11.7335 | 45.8324 |
| Uroteuthis edulis | S1 | 184 | 1.3747 | 18.2582 | 0.9145 | 0.6593 | 5.4968 | 0.6442 | 2.7017 | 0.7192 | 0.3736 | 0.3037 | 0.2635 | 0.4110 | 2.2845 | 0.5222 | 1.7487 | 0.3133 | 14.1473 | 46.9669 |
| Uroteuthis edulis | S5 | 186 | 1.1558 | 16.7953 | 0.6479 | 0.5191 | 4.7186 | 0.5283 | 2.3529 | 0.6918 | 0.3215 | 0.2904 | 0.2236 | 0.3528 | 2.7609 | 0.7557 | 2.3498 | 0.2929 | 16.9783 | 46.6748 |
| Uroteuthis edulis | S1 | 189 | 1.7786 | 16.9474 | 0.8087 | 0.7865 | 0.5862 | 2.8004 | 1.5950 | 0.7774 | 0.5634 | 0.5789 | 0.7411 | 1.4364 | 0.4341 | 0.1670 | 0.4336 | 0.4160 | 10.1597 | 40.4120 |
| Uroteuthis edulis | S1 | 193 | 2.2054 | 19.8626 | 0.2124 | 0.6189 | 2.4307 | 0.7063 | 1.8738 | 2.0726 | 0.7413 | 0.6880 | 0.6551 | 0.8138 | 4.1783 | 0.8104 | 5.5709 | 0.5404 | 13.2407 | 37.0366 |
| Uroteuthis edulis | S1 | 195 | 1.4240 | 17.6567 | 0.3241 | 0.6849 | 6.0254 | 0.4410 | 2.7258 | 0.6013 | 0.3585 | 0.2371 | 0.2048 | 0.2962 | 3.1646 | 0.5246 | 3.7223 | 0.2218 | 15.1607 | 41.9583 |
