# Supplementary material for: Fatty acid comparison of four sympatric loliginid squids in the northern South China Sea: Indication for their similar feeding strategy
Source: PLoS One. 2020 Jun 11;15(6):e0234250. doi: 10.1371/journal.pone.0234250 (PMC7289379; doi:10.1371/journal.pone.0234250)
Supplement: S1 Fig — (DOCX) [file pone.0234250.s008.docx]

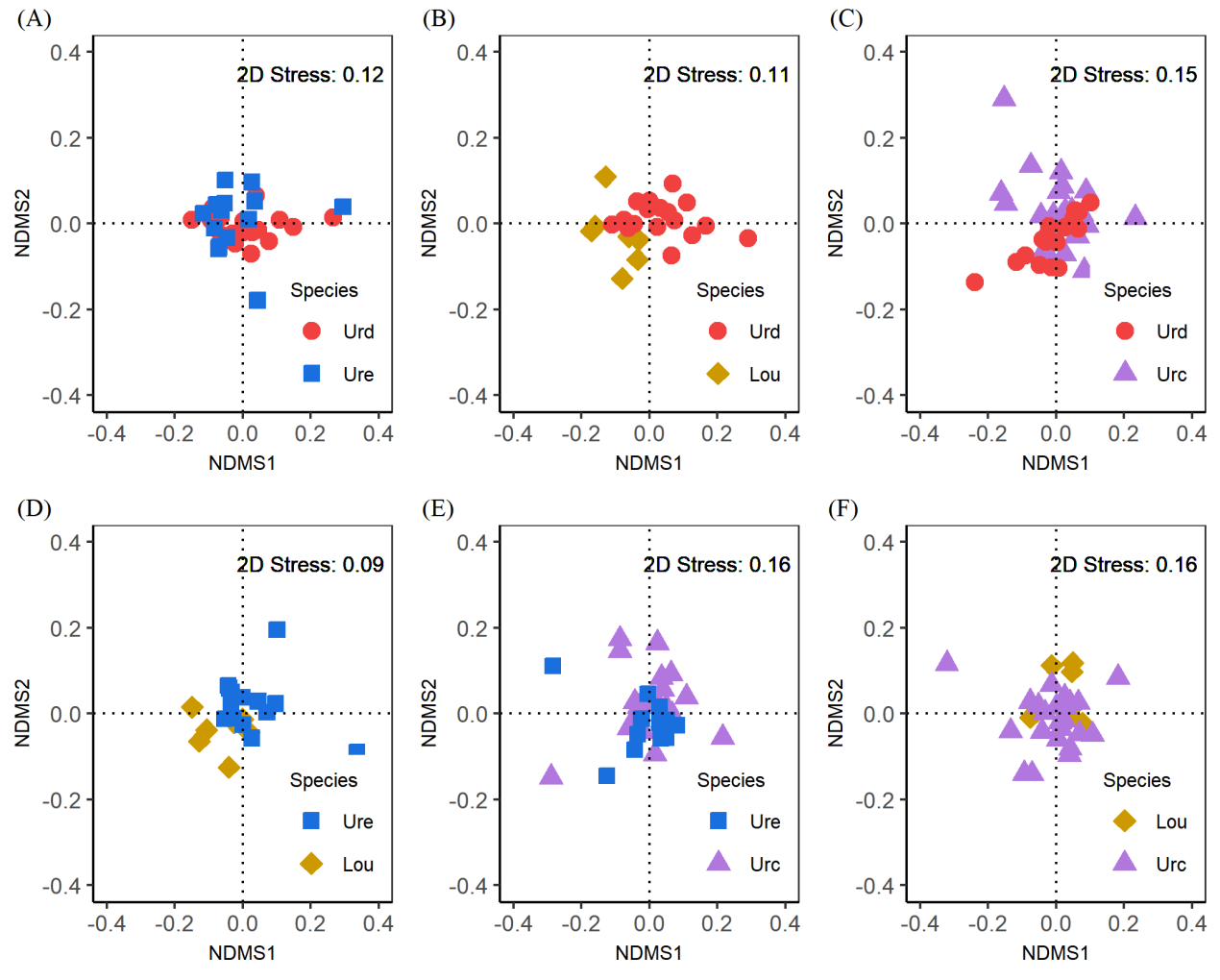


S1 Fig Non-metric multidimensional scaling (nMDS) ordination of fatty acid composition between each species pairing: Urd, *Uroteuthis duvaucelii*; Ure, *Uroteuthis edulis*; Lou, *Loliolus uyii*; Urc, *Uroteuthis chinensis*.
